# Proteome changes in the small intestinal mucosa of growing pigs with dietary supplementation of non-starch polysaccharide enzymes

**DOI:** 10.1186/s12953-016-0109-6

**Published:** 2017-01-10

**Authors:** Jize Zhang, Yang Gao, Qingping Lu, Renna Sa, Hongfu Zhang

**Affiliations:** 1 Institute of Grassland Research, Chinese Academy of Agricultural Sciences, Hohhot, 010010 People’s Republic of China; 2 State Key Laboratory of Animal Nutrition, Institute of Animal Sciences, Chinese Academy of Agricultural Sciences, Beijing, 100193 People’s Republic of China; 3 College of Animal Science and Technology, Jilin Agricultural University, Changchun, 130118 People’s Republic of China

**Keywords:** Non-starch polysaccharide enzymes, Small intestinal mucosa, Proteomics, Growing pigs

## Abstract

**Background:**

Non-starch polysaccharide enzymes (NSPEs) have long been used in monogastric animal feed production to degrade non-starch polysaccharides (NSPs) to oligosaccharides in order to promote growth performance and gastrointestinal (GI) tract health. However, the precise molecular mechanism of NSPEs in the improvement of the mammalian small intestine remains unknown.

**Methods:**

In this study, isobaric tags were applied to investigate alterations of the small intestinal mucosa proteome of growing pigs after 50 days of supplementation with 0.6% NSPEs (mixture of xylanase, β-glucanase and cellulose) in the diet. Bioinformatics analysis including gene ontology annotation was performed to determine the differentially expressed proteins. A protein fold-change of ≥ 1.2 and a *P*-value of < 0.05 were selected as thresholds.

**Results:**

Dietary supplementation of NSPEs improved the growth performance of growing pigs. Most importantly, a total of 90 proteins were found to be differentially abundant in the small intestinal mucosa between a control group and the NSPE group. Up-regulated proteins were related to nutrient metabolism (energy, lipids, protein and mineral), immunity, redox homeostasis, detoxification and the cell cytoskeleton. Down-regulated proteins were primarily related to transcriptional and translational regulation. Our results indicate that the effect of NSPEs on the increase of nutrient availability in the intestinal lumen facilitates the efficiency of nutrient absorption and utilization, and the supplementation of NSPEs in growing pigs also modulates redox homeostasis and enhances immune response during simulating energy metabolism due to a higher uptake of nutrients in the small intestine.

**Conclusions:**

These findings have important implications for understanding the mechanisms of NSPEs on the small intestine of pigs, which provides new information for the better utilization of this feed additive in the future.

**Electronic supplementary material:**

The online version of this article (doi:10.1186/s12953-016-0109-6) contains supplementary material, which is available to authorized users.

## Background

Many cereals such as soybean and wheat contain up to 15% non-starch polysaccharides (NSPs) in their outer or inner cell walls [1]. Monogastric animals lack enzymes to degrade the cell wall and NSP in these feeds. Thus, these anti-nutritive factors may interfere with digestion, nutrient absorption, and intestinal tract health by encapsuling starch and protein, as well as increase the viscosity of the chymus, which may elevate the proliferation of pathological bacteria in the small intestine and reduce the feed conversion ratio of monogastric livestock species [2–4].

The supplementation of exogenous enzymes such as xylanases and β-glucanases in pig diets may facilitate the hydrolysis of the main NSPs and increase the utilization of available raw materials [5, 6]. Adding exogenous enzymes to cereal diets improves both nutrient digestibility and growth performance in pigs [7, 8]. However, the exact molecular mechanisms of NSPEs, particularly in the gastrointestinal (GI) tract, are unknown [9]. There are several indications that exogenous enzymes may function in the GI tract of animals to aid digestion. The supplementation of NSPEs in the diets could increase the activities of certain types of digestive enzymes in vivo including protease, trypsin, and α-amylase [2, 4, 10]. These enzymes reduce the degradation of NSPs within the small intestine, thereby decreasing the viscosity of the digesta, which leads to a reduced bacterial load in the gut, especially potential pathogens [11]. Furthermore, the degradation of NSPs due to the supplementation of NSPEs promotes the higher availability of digestible nutrients such as energy substrates [12]. Additionally, the intestinal morphological structure and some physiological functions in animals benefit from the improvement of the changing intestinal environment due to the supplementation of NSPEs. Some research demonstrated that intestinal morphologies, including the villus height, the ratio of villus height to crypt depth, and the number of crypts and goblet cells, were changed due to the addition of xylanases alone or multiple enzymes [13, 14]. In addition to the effects of NSPEs observed on the GI tract, alterations of blood parameters related to the nutrient metabolism were also noted [15].

Previous studies reported that diet composition affected gene expression in animals [9, 16]. It is assumed that the improvement of the intestinal environment due to the supplementation of NSPEs in the diet may influence the gene expression and subsequent protein expression of epithelial-cell nutrient transporters in the GI tract mucosa, which has not been studied before. However, RNA editing and numerous options for posttranslational modifications should be taken into account [17, 18]. Hence, elucidation protein expression is important [19].

It is impractical to simultaneously measure all protein expression in the GI mucosa by classical method, such as western blotting. More research has yielded high throughput mass spectrometric proteomic technologies that can simultaneously detect hundreds of proteins [20, 21]. A proteomic analysis of the rat small intestinal proteome showed the presence of previously unrecognized proteins involved in various functions including the absorption and transport of nutrients and the maintenance of cell structure, as well as intestinal molecular chaperones [22]. There remains a great need to pursue proteomic technology to elucidate the beneficial effects of NSPEs in the GI tract mucosa. Therefore, we utilized a label-based iTRAQ (isobaric tags for relative and absolute quantitation) method, followed by LC-MS/MS, to quantitate proteins that are differentially induced in the small intestinal mucosa of growing pigs supplemented with NSPEs in the diet.

## Methods

### Enzyme preparation

The NSP enzyme mixture preparation supplemented in the diet was provided by the State Key Laboratory of Animal Nutrition, Institute of Animal Sciences, Chinese Academy of Agricultural Sciences (Beijing, China); the mixture contained 7 × 10^5^ U/g xylanase activity (EC 3.2.1.8), 1 × 10^5^ U/g β-glucanase activity (EC 3.2.1.6), and 9000 U/g cellulase activity (EC 3.2.1.4). The activities of the enzymes used in the present study was measured according the methods mentioned in previous research [23].

### Animals and treatments

Forty-eight crossbred (Duroc × Landrace × Large White) growing pigs had similar initial body weights (39.18 ± 0.98 kg); the pigs were obtained from a commercial farm in Beijing (Shunliang pig farm, Beijing). The pigs were randomly divided into two groups according to their littermates, sex and mean initial body weights with four replicates in each group and six pigs in each replicate (half females and half males). The following two groups were a control group (CTRL, basal diet) and a treatment group (NSPE, basal diet + 0.6% NSP enzymes). The amount of NSPEs supplementation in the present study was based on the previous results from our group [24]. Both diets were formulated to meet NRC (2012) recommendations (Table 1). All pigs were kept in eight adjacent pens covered in a fermentation bed facility. Feed and water were provided *ad libitum* during the 50 day experimental period. The individual pig weight and feed intake were recorded at the initiation and the termination of the experiment for the measurement of the average daily gain (ADG), average daily feed intake (ADFI) and feed conversion ratio (FCR). All procedures involving animals were evaluated and approved by the Animal Ethics Committee of the Institute of Animal Sciences, Chinese Academy of Agricultural Sciences.

**Table 1:** Composition of the basal diet and calculated proximate composition of the diet

Ingredients	Proportion (%)^a^
Corn	70.70
Soybean meal	19.82
Soybean oil	2.10
Wheat bran	5.00
Limestone	0.51
Calcium hydrophosphate	0.56
L-Lysine	0.01
Sodium chloride	0.30
Premix^b^	1.00
Total	100
Nutrient	
ME	13.65 (MJ/kg)
Ether extract (EE)	4.82
Crude protein (CP)	15.50
Calcium	0.50
Total phosphorus	0.45
Available phosphorus	0.24
Total lysine	0.75
Total methionine	0.25

^a^All data is expressed in g/kg dry weight except for metabolizable energy (ME) in MJ/kg. The amounts of nutrient were estimated based on the NRC 11th ed. swine feedstuff composition table

^b^Providing the following (g/kg fresh weight), Vitamin A, 8250 IU; Vitamin D_3_: 825 IU; Vitamin E: 40 IU; Vitamin K_3_, 4.0 mg; Vitamin B_1_, 1.0 mg; Vitamin B_2_, 5.0 mg; Vitamin B_6_, 2.0 mg; Vitamin B_12_, 25 μg; choline chloride, 600 mg; nicotinic acid, 35 mg; folic acid, 2.0 mg; biotin, 4.0 mg; Cu, 50.0 mg; Fe, 80.0 mg; Zn, 100.0 mg; Mn, 25.0 mg; Se, 0.15 mg; I, 0.5 mg

### Sample collection

At the end of the experiment (Day 50), all pigs were weighted after 12 h of fasting. One pig per replicate, a total of eight pigs (*n* = 8), were sacrificed by CO_2_ asphyxiation and then exsanguinated. Blood samples were obtained from the cervical vein by syringe before sacrifice. The whole blood was centrifuged at 2000 g for 30 min at 4 °C, followed by centrifugation at 400 g for 10 min at 4 °C. Then, the resulting supernatant was collected as sera samples, which were stored at −20 °C for further analysis. A 20-cm tissue section was rapidly excised at 50% of the length of the small intestine, rinsed with cold phosphate buffer saline, and blotted dry on paper. Mucosa from this small intestine section was sequentially obtained by careful scraping of the mucosal layer using a glass microscope slide as previously described [25]. Then, the collected mucosal samples were snap-frozen in liquid nitrogen and stored at −80 °C for proteomic analysis.

### Serum biochemical analyses

Important serum biochemical parameters, including alanine aminotransferase (ALT), aspartate aminotransferase (AST), total protein (TP), alkaline phosphatase (ALP), glucose (GLU), and creatine kinase (CK), were analyzed using an automatic biochemical analyzer (Hitachi 7020, Tokyo, Japan). Serum levels of total superoxide dismutase (T-SOD) and immunoglobulin G (IgG) were measured using a corresponding kit (Nanjing Jiancheng Bioengineering Institute, Nanjing, China) according to the manufacturer’s instructions.

### Protein extraction and sample preparation

Small intestinal mucosa samples (500 μg) were ground in liquid nitrogen using a Dounce glass grinder. Grinded powder was precipitated with 10% trichloroacetic acid (TCA) (w/v) and 90% ice-cold acetone at −20 °C for 2 h. The precipitate was obtained by centrifugation at 20,000 g for 30 min at 4 °C and subsequently washed with ice-cold acetone. Then, the precipitate was lysed in lysis buffer [8 M urea, 30 mM 4-(2-hydroxyethyl)-1-piperazineethanesulfonic acid (HEPES), 1 mM phenylmethanesulfonyl fluoride (PMSF), 2 mM ethylene diamine tetraacetic acid (EDTA), and 10 mM dithiothreitol (DTT)]. The crude tissue extracts were centrifuged to remove the remaining debris. The tissue lysates were reduced for 1 h at 56 °C in a water bath using 10 mM DTT and then alkylated with 55 mM iodoacetamide for 1 h in the dark. Afterwards, the lysates were precipitated by adding four volumes of pre-chilled acetone. The pellets were then washed three times with pre-chilled pure acetone and resuspended in the buffer (50% TEAB and 0.1% SDS). The centrifugation was repeated to remove the undissolved pellets. Subsequently, protein quantitation was determined using a Bio-Rad Bradford Protein Assay Kit (Hercules, CA, USA). Each sample was digested with modified sequence grade trypsin (Promega Corporation, Madison, WI) at a 1: 30 ratio (3.3 μg trypsin : 100 μg target) overnight at 37 °C. Each isobaric tag (113, 114, 115, 116, 117, 118, 119, and 121) was solubilized in 70 μL isopropanol and then added to each respective sample (4 samples per group). Incubation continued for 2 h at room temperature.

### Strong cation exchange chromatography

The strong cation exchange fractionation was performed according to a previous report [26] with slight modification. Briefly, 800 μg of labeled sample was loaded onto a strong cation exchange column (Phenomenex Luna SCX 100A) installed in an Agilent 1100 (Santa Clara, CA) system and equilibrated with buffer A (25% acetonitrile and 10 mM KH_2_PO_4_, pH 3.0). The peptides were separated by a linear gradient of buffer B (25% acetonitrile, 2 M KCl and 10 mM KH_2_PO_4_, pH 3.0) according to this procedure (increasing to 5% after 41 min, 50% after 66 min and 100% after 71 min with a flow rate of 1 ml/min). Elution was monitored by setting the absorbance at 214 nm. A total of 10 fractions were obtained, then desalted with a Strata X C18 column (Phenomenex) and dried under a vacuum. The pellets were resuspended by adding 0.1% formic acid before the LC-MS/MS run.

### Mass spectrometry

LC-MS/MS was conducted according to a previous report [27], and the detailed process and parameters are shown in Additional file 1.

### Data processing and protein quantification

All the detailed parameters are shown in the Supporting Information (Additional file 1). MS/MS data for iTRAQ protein identification and quantitation were analyzed using Proteome Discover 1.3 (Thermo Fisher Scientific, Bremen, Germany) and in-house MASCOT software (Matrix Science, London, UK; Version 2.3.0) against the database Uniprot_pig (Apr. 11th, 2014). Median ratio normalization was performed in intra-sample channels to normalize each channel across all proteins. Protein quantitative ratios for each iTRAQ labeled sample were obtained, using a sample in the control group (sample tagged with 113) as the denominator. Quantitative ratios were then log transformed to base two and presented as the fold change relative to the denominator in the control group for final quantitative testing. Differentially expressed proteins were identified using Student’s *t*-test corrected for multiple testing using the Benjamini and Hochberg correction [21, 25, 28, 29]. Based on above the relative quantification, statistical analysis, and a number of previous reports regarding to iTRAQ experiments [29–31], we set a 1.2-fold change or greater as the threshold for differentially expressed proteins.

### Bioinformatics analysis and validation of protein expression

The databases and software for bioinformatics analysis are shown in Additional file 1. Real-time qPCR was used to verify six small intestinal mucosal proteins of differential abundance at the mRNA level. All detailed procedures are described in the Supporting Information (Additional file 1). The primer sequences used in this study are shown in Additional file 2: Table S1.

### Statistical analysis

The data for growth parameters, serum parameters, and gene expression were analyzed by one-way ANOVA using block as a covariate (SAS Version 9.2, SAS institute Inc., Cary, NC) according the previous studies [21, 31], and a *t*-test was used for independent samples in MS data analysis. A group difference was assumed statistically significant when *P* < 0.05.

## Results

### Growth performance of growing pigs

During the entire experimental period (50 days), NSPE pigs had 15.5% greater ADG (*P* < 0.05) compared with the control group; however, the ADFI between the two groups was not significantly different (*P* > 0.05). It is notable that pig fed NSPEs had an 8.7% greater FCR compared with the control group (*P* < 0.05; Table 2).

**Table 2:** Effects of NSP enzymes on growth performance of growing pigs

	Groups		
	Control	Treatment	*P* value
Initial weight (kg)	38.80 ± 0.99	39.55 ± 0.63	0.1245
Final weight (kg)	74.04 ± 1.77^b^	78.42 ± 1.06^a^	0.0318
ADG (kg/d)^c^	0.71 ± 0.05^b^	0.82 ± 0.05^a^	0.0437
ADFI (kg/d)^d^	1.97 ± 0.09	2.07 ± 0.06	0.0423
FCR (kg feed/kg weight gain)^e^	2.77 ± 0.02^a^	2.53 ± 0.03^b^	0.0352

^a, b^ Values within a column having different superscript letters indicate a significant difference at *P* < 0.05. Numbers are mean ± S.D. (*n* = 24 for ADG; *n* = 4 for ADFI and FCR)

^c^ ADG = average daily gain

^d^ ADFI = average daily feed intake

^e^ FCR = feed conversion ratio

### Serum parameters of growing pigs

In NSPE pigs, serum concentration of CK was significantly lower (*P* < 0.05) than the control group (Table 3). Furthermore, the serum concentrations of T-SOD, IgG, and glucose were significantly elevated compared with the control group (*P* < 0.05) (Table 3). Serum levels of TP, ALT and AST were similar between the two groups (Table 3).

**Table 3:** Effect of NSPEs on serum biochemical parameters of growing pigs

	Groups		
	Control	Treatment	*P* value
ALT (IU/L)^c^	49.01 ± 7.96	49.00 ± 9.30	0.4768
AST (IU/L)^d^	79.60 ± 10.70	63.80 ± 16.05	0.2240
TP (mmol/L)^e^	67.31 ± 5.44	69.50 ± 2.44	0.5331
ALP (U/L)^f^	131.83 ± 36.14	126.40 ± 22.06	0.2565
GLU (mmol/L)^g^	6.37 ± 2.24^b^	9.73 ± 2.34^a^	0.0479
T-SOD (U/mL)^h^	61.55 ± 2.67^b^	67.44 ± 3.64^a^	0.0002
CK (U/L)^i^	3117 ± 274^a^	2188 ± 218^b^	0.0089
IgG (g/L)^j^	3.19 ± 0.16^b^	3.43 ± 0.20^a^	0.0392

^a, b^Values within a column not sharing a common superscript letter indicate significant difference at *P* < 0.05. Numbers are means ± S.D. (*n* = 4)

^c^ALT = alanine aminotransferase

^d^AST = aspartate aminotransferase

^e^TP = total protein

^f^ALP = alkaline phosphatase

^g^GLU = glucose

^h^T-SOD = total superoxide dismutase

^i^CK = creatine kinase

^j^IgG = immunoglobulin G

### Identification and comparison of proteins of differential abundance

Using iTRAQ analysis, a total of 2634 proteins were identified within the FDR (false discovery rate) of 1% (Additional file 3: Table S2). Following statistical analysis, 104 proteins were found to be differentially expressed in the small intestinal mucosa between CTRL and NSPE pigs, with 43 up-regulated and 61 down-regulated (Additional file 4: Table S3).

**Fig. 1:**
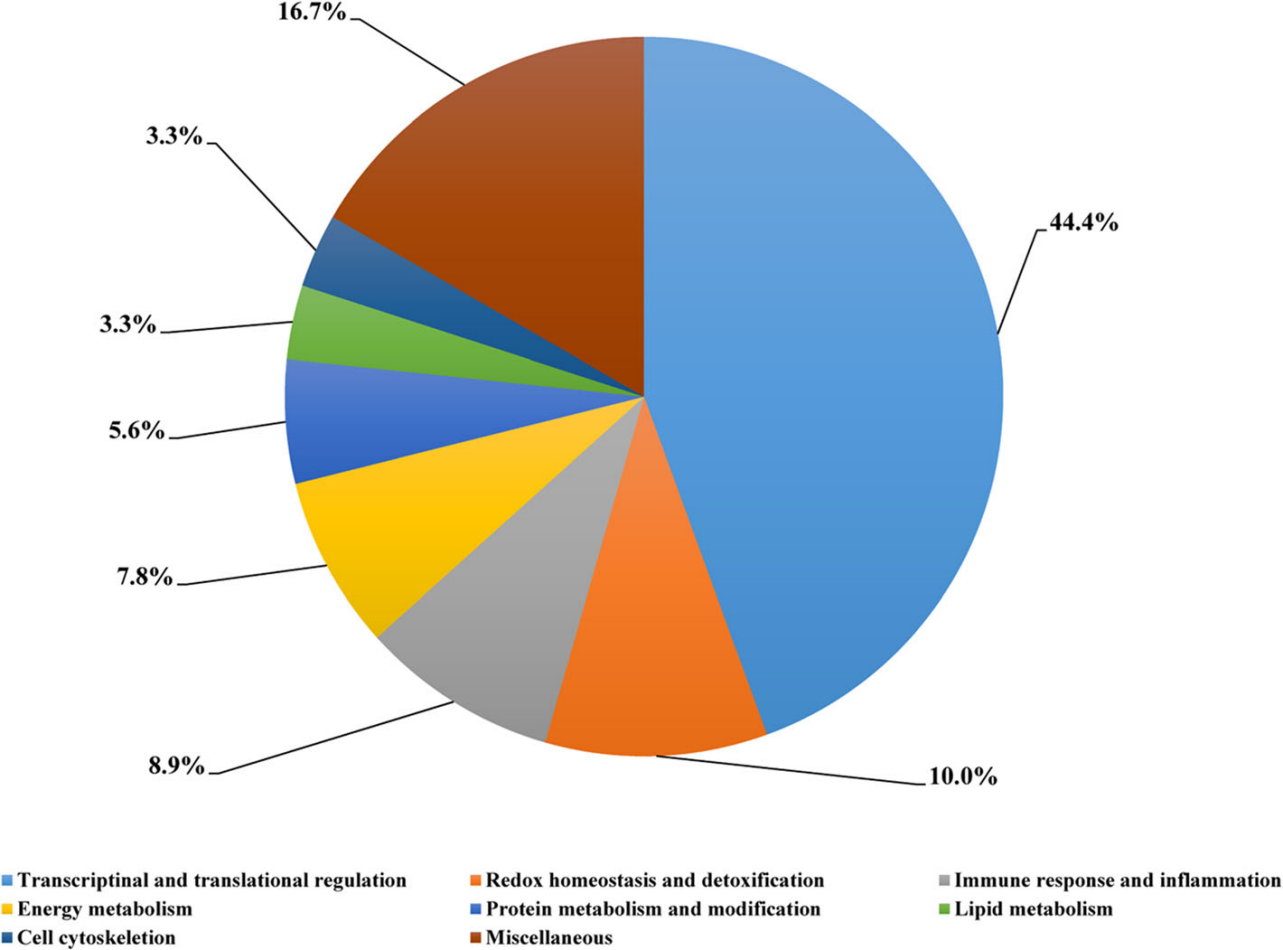
Functional classification of the proteins of differential abundance identified from the small intestinal mucosa of growing pigs supplemented with NSPE

A total of 90 proteins of differential abundance were grouped into eight classes based on putative functions: transcriptional and translational regulation (44.4%), miscellaneous (16.7%), redox homeostasis and detoxification (10.0%), immune response and inflammation (8.9%), energy metabolism (7.8%), protein metabolism and modification (5.6%), lipid metabolism (3.3%), and cell cytoskeleton (3.3%) (Fig. 1). Those related to transcriptional and translational regulation, redox homeostasis, and immune response were predominant, accounting for approximately 63% of the differentially expressed proteins. A comparison of proteins of differential abundance with functional groupings between the two groups indicated that a smaller number of protein species were up-regulated in NSPE pigs (36 versus 54) (Table 4).

**Table 4:** List of differentially expressed proteins in small intestinal mucosal samples from treatment group and control group

Accession^a^	Description^b^	Gene symbol	Score^c^	Pep. No^d^	Log_2_ fold change	*P*-value^e^	Biological process GO term
Transcriptional and translational regulation							
F1S419	Uncharacterized protein OS = Sus scrofa GN = SF3B3 PE = 4 SV = 2 - [F1S419_PIG]	None	85.61	3	−0.37	0.0007	RNA binding
K9J4V0	U5 small nuclear ribonucleoprotein 200 kDa helicase OS = Sus scrofa GN = SNRNP200 PE = 2 SV = 1 - [K9J4V0_PIG]	SNRNP200	248.18	9	−0.32	0.0012	Nucleic acid binding
F2Z5Q6	40S ribosomal protein S6 (Fragment) OS = Sus scrofa GN = RPS6 PE = 3 SV = 2 - [F2Z5Q6_PIG]	RPS6	140.42	4	−0.81	0.0013	Structural constituent of ribosome
F1SD96	Uncharacterized protein (Fragment) OS = Sus scrofa GN = RAD23A PE = 4 SV = 1 - [F1SD96_PIG]	RAD23A	85.25	3	1.06	0.0026	Nucleotide excision repair
F1S8K5	Uncharacterized protein OS = Sus scrofa GN = SUPT16H PE = 4 SV = 1 - [F1S8K5_PIG]	SUPT16H	35.43	2	−0.40	0.0028	RNA binding
F1RZH4	Uncharacterized protein OS = Sus scrofa PE = 4 SV = 1 - [F1RZH4_PIG]	ADAM10	32.67	1	−0.82	0.0048	Structural constituent of ribosome
F1SD98	Uncharacterized protein OS = Sus scrofa GN = TRMT1 PE = 4 SV = 2 - [F1SD98_PIG]	TRMT1	27.72	1	−0.30	0.0065	Poly(A) RNA binding
I3LHZ6	Uncharacterized protein OS = Sus scrofa GN = DHX9 PE = 4 SV = 1 - [I3LHZ6_PIG]	DHX9	994.71	27	−0.30	0.0075	ATP-dependent RNA helicase activity
F1SDV7	Uncharacterized protein (Fragment) OS = Sus scrofa GN = TOP1 PE = 4 SV = 1 - [F1SDV7_PIG]	TOP1	99.93	4	−0.44	0.0075	DNA binding
P62802	Histone H4 OS = Sus scrofa PE = 1 SV = 2 - [H4_PIG]	None	358.11	7	−0.67	0.0103	DNA binding
F1S1V1	Uncharacterized protein OS = Sus scrofa GN = SSB PE = 4 SV = 2 - [F1S1V1_PIG]	SSB	196.5	6	−0.73	0.0111	Nucleotide binding
F1RS45	DNA topoisomerase 2 OS = Sus scrofa PE = 3 SV = 2 - [F1RS45_PIG]	TOP2B	116.62	6	−0.27	0.0117	DNA binding
F1S1X3	Uncharacterized protein OS = Sus scrofa GN = NARS PE = 3 SV = 2 - [F1S1X3_PIG]	NARS	262.65	7	−0.30	0.0119	Nucleotide binding
F2Z576	Histone H3 OS = Sus scrofa GN = LOC100525821 PE = 2 SV = 1 - [F2Z576_PIG]	HIST1H3E	159.44	6	−0.77	0.0120	DNA binding
Q29194	Ribosomal protein S2 (Fragment) OS = Sus scrofa PE = 2 SV = 1 - [Q29194_PIG]	None	46.59	1	−0.45	0.0138	Structural constituent of ribosome
I3LFV4	Uncharacterized protein OS = Sus scrofa GN = YBX1 PE = 4 SV = 1 - [I3LFV4_PIG]	YBX1	157.89	4	0.41	0.0148	DNA repair
I3LIN8	Histone H2A OS = Sus scrofa GN = H2AFY PE = 3 SV = 1 - [I3LIN8_PIG]	H2AFY	224.89	6	−0.52	0.0149	Chromatin DNA binding
B0FWK5	Ribosomal protein L5 OS = Sus scrofa GN = RPL5 PE = 2 SV = 1 - [B0FWK5_PIG]	RPL5	178.57	8	−0.34	0.0165	Structural constituent of ribosome
I3LCI4	Uncharacterized protein OS = Sus scrofa GN = ZFR PE = 4 SV = 1 - [I3LCI4_PIG]	ZFR	41.93	2	−0.28	0.0167	Poly(A) RNA binding
F1S8A5	Uncharacterized protein OS = Sus scrofa GN = MRPS26 PE = 4 SV = 1 - [F1S8A5_PIG]	MRPS26	38.63	1	−0.36	0.0181	Poly(A) RNA binding
A5GFY4	Negative elongation factor D OS = Sus scrofa GN = NELFCD PE = 3 SV = 1 - [NELFD_PIG]	NELFCD	43.67	1	−0.32	0.0189	Negative regulation of transcription
F1S5A8	Uncharacterized protein OS = Sus scrofa GN = DHX15 PE = 4 SV = 1 - [F1S5A8_PIG]	DHX15	259.43	8	−0.26	0.0198	ATP-dependent RNA helicase activity
F1RRG9	Uncharacterized protein OS = Sus scrofa GN = SMARCA5 PE = 4 SV = 1 - [F1RRG9_PIG]	SMARCA5	99.44	3	−0.39	0.0201	DNA binding
F1RGP1	Uncharacterized protein OS = Sus scrofa GN = MYBBP1A PE = 4 SV = 1 - [F1RGP1_PIG]	MYBBP1A	445.89	12	−0.50	0.0208	Poly(A) RNA binding
F2Z5Q8	Uncharacterized protein OS = Sus scrofa GN = LOC100519675 PE = 4 SV = 1 - [F2Z5Q8_PIG]	RPL35A	57.33	2	−0.45	0.0209	Structural constituent of ribosome
I3L7T6	Histone H2A OS = Sus scrofa GN = H2AFX PE = 3 SV = 1 - [I3L7T6_PIG]	H2AFX	357.7	7	−0.56	0.0231	DNA binding
F1SMZ9	Uncharacterized protein (Fragment) OS = Sus scrofa GN = SF3B1 PE = 4 SV = 2 - [F1SMZ9_PIG]	SF3B1	267.33	9	−0.26	0.0245	mRNA binding
F2Z5K9	Histone H3 OS = Sus scrofa GN = LOC100622412 PE = 3 SV = 1 - [F2Z5K9_PIG]	LOC100622412	178.75	6	−0.76	0.0270	DNA binding
P53027	60S ribosomal protein L10a (Fragment) OS = Sus scrofa GN = RPL10A PE = 2 SV = 3 - [RL10A_PIG]	RPL10A	154.25	5	−0.34	0.0272	RNA binding
K9IVG8	DEAD (Asp-Glu-Ala-Asp) box helicase 21 OS = Sus scrofa GN = DDX21 PE = 2 SV = 1 - [K9IVG8_PIG]	DDX21	44.62	1	−0.38	0.0292	RNA binding
F2Z554	Uncharacterized protein OS = Sus scrofa GN = RPL30 PE = 3 SV = 1 - [F2Z554_PIG]	RPL30	105.87	4	−0.26	0.0323	RNA binding
Q29195	60S ribosomal protein L10 OS = Sus scrofa GN = RPL10 PE = 2 SV = 3 - [RL10_PIG]	RPL10	105.8	4	−0.39	0.0350	Structural constituent of ribosome
P67985	60S ribosomal protein L22 OS = Sus scrofa GN = RPL22 PE = 2 SV = 2 - [RL22_PIG]	RPL22	113.83	3	−0.48	0.0355	Structural constituent of ribosome
I7GF95	Guanine nucleotide binding protein-like 1 OS = Sus scrofa GN = GNL1 PE = 4 SV = 1 - [I7GF95_PIG]	GNL1	58.56	1	−0.35	0.0371	Ribosome biogenesis
F1S8L9	Uncharacterized protein OS = Sus scrofa GN = HNRNPU PE = 4 SV = 2 - [F1S8L9_PIG]	HNRNPU	883.61	23	−0.34	0.0377	Poly(A) RNA binding
Q53DY5	Histone H1.3-like protein OS = Sus scrofa GN = LOC595122 PE = 2 SV = 1 - [Q53DY5_PIG]	HIST1H1D	251.92	7	1.29	0.0384	Chromatin DNA binding
F1S2G3	Uncharacterized protein (Fragment) OS = Sus scrofa GN = TBCA PE = 4 SV = 1 - [F1S2G3_PIG]	TBCA	78.18	2	0.31	0.0389	Poly(A) RNA binding
F2Z5P1	Histone H2A (Fragment) OS = Sus scrofa GN = H2AFV PE = 3 SV = 1 - [F2Z5P1_PIG]	LOC100512448	256.74	5	−0.43	0.0427	DNA binding
F2Z553	Uncharacterized protein OS = Sus scrofa GN = EIF1 PE = 4 SV = 1 - [F2Z553_PIG]	EIF1	103.66	2	0.82	0.0437	Translation initiation factor activity
F2Z5L5	Histone H2A OS = Sus scrofa GN = HIST2H2AC PE = 3 SV = 1 - [F2Z5L5_PIG]	HIST2H2AC	322.1	5	−0.62	0.0448	DNA binding
Redox homeostasis and detoxification							
F1SKJ2	Uncharacterized protein OS = Sus scrofa GN = TXN2 PE = 4 SV = 1 - [F1SKJ2_PIG]	TXN2	29.86	1	0.39	0.0043	Cell redox homeostasis
F1SGS9	Catalase OS = Sus scrofa GN = CAT PE = 3 SV = 1 - [F1SGS9_PIG]	CAT	923.56	23	0.58	0.0151	Protect cells from the toxic effects of hydrogen peroxide
I3LDJ8	Uncharacterized protein OS = Sus scrofa PE = 3 SV = 1 - [I3LDJ8_PIG]	None	303.51	10	0.77	0.0202	Oxidoreductase activity
P12309	Glutaredoxin-1 OS = Sus scrofa GN = GLRX PE = 1 SV = 2 - [GLRX1_PIG]	GLRX	277.83	6	0.64	0.0208	Cell redox homeostasis
F1SCF9	Uncharacterized protein (Fragment) OS = Sus scrofa GN = TECR PE = 4 SV = 2 - [F1SCF9_PIG]	TECR	38.34	1	−0.37	0.0242	Oxidoreductase activity
A5J2A8	Thioredoxin (Fragment) OS = Sus scrofa GN = TRX PE = 4 SV = 1 - [A5J2A8_PIG]	TRX	128.36	3	0.34	0.0303	Cell redox homeostasis
F1SMY1	Uncharacterized protein OS = Sus scrofa GN = TMX3 PE = 4 SV = 2 - [F1SMY1_PIG]	TMX3	39.1	2	0.30	0.0345	Cell redox homeostasis
P16549	Dimethylaniline monooxygenase [N-oxide-forming] 1 OS = Sus scrofa GN = FMO1 PE = 1 SV = 3 - [FMO1_PIG]	FMO1	39.55	2	1.64	0.0084	Oxidative metabolism of a variety of xenobiotics
P04178	Superoxide dismutase [Cu-Zn] OS = Sus scrofa GN = SOD1 PE = 1 SV = 2 - [SODC_PIG]	SOD1	459.04	9	0.35	0.0424	Superoxide dismutase activity
Immune response and inflammation							
A3FJ41	MHC class I antigen (Fragment) OS = Sus scrofa GN = SLA-1 PE = 4 SV = 1 - [A3FJ41_PIG]	SLA-1	120.03	5	0.35	0.0050	Immune response
F1RGC8	Uncharacterized protein OS = Sus scrofa GN = NLRP6 PE = 4 SV = 3 - [F1RGC8_PIG]	NLRP6	119.58	4	−0.32	0.0061	Activation of NF-κB
F1RFM7	Uncharacterized protein OS = Sus scrofa GN = AIMP2 PE = 4 SV = 1 - [F1RFM7_PIG]	AIMP2	232.75	6	−0.29	0.0076	Metabolism of xenobiotics
A2SZV5	Tax1 binding protein 3 (Fragment) OS = Sus scrofa PE = 4 SV = 1 - [A2SZV5_PIG]	None	55.14	1	0.29	0.0133	Negative regulation of NF-κB
B8XX91	DNA-dependent activator of IFN-regulatory factor OS = Sus scrofa GN = DAI PE = 2 SV = 1 - [B8XX91_PIG]	DAI	100.5	4	0.70	0.0137	Innate immune responses
Q8WNQ7	N-acetylgalactosamine-6-sulfatase OS = Sus scrofa GN = GALNS PE = 2 SV = 1 - [GALNS_PIG]	GALNS	52.52	1	0.60	0.0311	Degradation of the glycosaminoglycans keratan sulfate
B8XTR8	Granzyme H OS = Sus scrofa GN = gzmH PE = 2 SV = 1 - [B8XTR8_PIG]	gzmH	168.84	6	−0.67	0.0272	Serine-type endopeptidase activity
A5GFQ5	Protein canopy homolog 3 OS = Sus scrofa GN = CNPY3 PE = 3 SV = 1 - [CNPY3_PIG]	CNPY3	40.13	2	−0.63	0.0376	Receptor binding for proper TLR folding
Energy metabolism							
Q1ACV5	Transporter associated with antigen processing 1 OS = Sus scrofa PE = 2 SV = 1 - [Q1ACV5_PIG]	None	298.67	7	−0.32	0.0030	Triggers ATP hydrolysis
F1RIG0	Uncharacterized protein (Fragment) OS = Sus scrofa PE = 4 SV = 1 - [F1RIG0_PIG]	None	47.28	2	−0.27	0.0169	ATP binding
Q7SIB7	Phosphoglycerate kinase 1 OS = Sus scrofa GN = PGK1 PE = 1 SV = 3 - [PGK1_PIG]	PGK1	850.39	23	0.30	0.0160	Conversion of 1,3-diphosphoglycerate to 3-phosphoglycerate
H9BYW2	Acyl-coenzyme A oxidase OS = Sus scrofa GN = ACOX1 PE = 2 SV = 1 - [H9BYW2_PIG]	ACOX1	370.35	10	0.91	0.0200	Fatty acid beta-oxidation
I3LEN7	Uncharacterized protein OS = Sus scrofa GN = ALDH1L1 PE = 3 SV = 1 - [I3LEN7_PIG]	ALDH1L1	49.04	2	0.40	0.0245	Formate oxidation
F1S0Y8	Uncharacterized protein OS = Sus scrofa GN = ADH4 PE = 3 SV = 2 - [F1S0Y8_PIG]	ADH4	40.7	2	0.67	0.0309	Oxidation of long-chain aliphatic alcohols
A7UIU7	ATP citrate lyase OS = Sus scrofa GN = ACL PE = 2 SV = 1 - [A7UIU7_PIG]	ACL	468.98	14	−0.38	0.0374	ATP binding
Protein metabolism and modification							
F1RIF3	Uncharacterized protein OS = Sus scrofa GN = FAH PE = 4 SV = 1 - [F1RIF3_PIG]	FAH	38.37	2	0.39	0.0010	Catabolism of the amino acid phenylalanine
Q9GK25	Peptidyl-prolyl cis-trans isomerase (Fragment) OS = Sus scrofa PE = 2 SV = 1 - [Q9GK25_PIG]	None	266.1	7	1.43	0.0025	Accelerate the folding of proteins
I3L739	Uncharacterized protein OS = Sus scrofa GN = JMJD6 PE = 4 SV = 1 - [I3L739_PIG]	JMJD6	39.99	1	−0.29	0.0193	Protein hydroxylases
I3LK37	Uncharacterized protein (Fragment) OS = Sus scrofa PE = 3 SV = 1 - [I3LK37_PIG]	GALNT7	33.39	2	−0.30	0.0248	Protein glycosylation
F1RNR6	4-hydroxyphenylpyruvate dioxygenase OS = Sus scrofa GN = HPD PE = 3 SV = 2 - [F1RNR6_PIG]	HPD	31	1	0.35	0.0391	Aromatic amino acid family metabolic process
Lipid metabolism							
I3LM15	Uncharacterized protein OS = Sus scrofa GN = AGPS PE = 4 SV = 1 - [I3LM15_PIG]	AGPS	48.77	1	−0.36	0.0019	Lipid biosynthetic process
Q9GJX2	Diazepam binding inhibitor (Fragment) OS = Sus scrofa GN = DBI PE = 2 SV = 1 - [Q9GJX2_PIG]	DBI	80.13	3	0.91	0.0057	Long-chain fatty acyl-CoA binding, triglyceride metabolic process
P27917	Apolipoprotein C-III OS = Sus scrofa GN = APOC3 PE = 1 SV = 2 - [APOC3_PIG]	APOC3	226.39	7	0.78	0.0241	High-density lipoprotein particle receptor binding
Cell cytoskeleton							
P10668	Cofilin-1 OS = Sus scrofa GN = CFL1 PE = 1 SV = 3 - [COF1_PIG]	CFL1	704.02	15	0.31	0.0059	Cytoskeleton organization
Q5G6W0	Cofilin-2 (Fragment) OS = Sus scrofa PE = 2 SV = 1 - [Q5G6W0_PIG]	CFL1	48.67	2	0.43	0.0073	Cytoskeleton organization
B5APV0	Actin-related protein 2/3 complex subunit 5 OS = Sus scrofa GN = ARPC5 PE = 2 SV = 1 - [B5APV0_PIG]	ARPC5	170.99	6	0.30	0.0167	Structural constituent of cytoskeleton
Miscellaneous							
Q9TSA7	Calmodulin (Fragments) OS = Sus scrofa PE = 4 SV = 1 - [Q9TSA7_PIG]	None	108.72	4	1.11	0.0008	Calcium ion binding
K7GKQ1	Uncharacterized protein OS = Sus scrofa GN = RAB9A PE = 3 SV = 1 - [K7GKQ1_PIG]	RAB9A	26.6	1	−0.40	0.0071	Cytoskeletal signaling
F1RKI3	Uncharacterized protein OS = Sus scrofa GN = HINT1 PE = 4 SV = 1 - [F1RKI3_PIG]	HINT1	80.55	3	0.32	0.0073	Tumor suppressing
I3LSY0	Uncharacterized protein OS = Sus scrofa GN = ACSM4 PE = 4 SV = 1 - [I3LSY0_PIG]	ACSM4	21.13	1	0.86	0.0179	Catalytic activity
D0G6R8	Phosphatidate cytidylyltransferase OS = Sus scrofa GN = CDS2 PE = 2 SV = 1 - [D0G6R8_PIG]	CDS2	33.01	1	−0.39	0.0192	Synthesis of phosphatidylglycerol
Q95332	Betaine--homocysteine S-methyltransferase 1 (Fragment) OS = Sus scrofa GN = BHMT PE = 1 SV = 3 - [BHMT1_PIG]	BHMT	110.41	4	1.10	0.0193	Regulation of homocysteine metabolism
F1RS34	Uncharacterized protein OS = Sus scrofa GN = GAPVD1 PE = 4 SV = 2 - [F1RS34_PIG]	GAPVD1	22.69	1	−0.40	0.0207	Signal transduction
F1ST01	Uncharacterized protein OS = Sus scrofa GN = SELENBP1 PE = 4 SV = 1 - [F1ST01_PIG]	SELENBP1	936.42	22	0.33	0.0209	Selenium binding
Q9TV62	Myosin-4 OS = Sus scrofa GN = MYH4 PE = 2 SV = 1 - [MYH4_PIG]	MYH4	192.94	7	−0.83	0.0336	Motor activity
F1RN91	Uncharacterized protein (Fragment) OS = Sus scrofa PE = 4 SV = 2 - [F1RN91_PIG]	MYO18A	35.04	2	0.28	0.0355	Cell migration
F1RPC8	Uncharacterized protein OS = Sus scrofa GN = CRYM PE = 4 SV = 2 - [F1RPC8_PIG]	CRYM	59.33	2	0.49	0.0392	Thyroid hormone binding
F2Z5W6	Uncharacterized protein OS = Sus scrofa GN = LAMTOR1 PE = 4 SV = 1 - [F2Z5W6_PIG]	LAMTOR1	26.54	1	−0.37	0.0410	Guanyl-nucleotide exchange factor activity
Q29069	Myosin light chain OS = Sus scrofa PE = 2 SV = 2 - [Q29069_PIG]	None	58.61	3	−0.38	0.0458	Calcium ion binding
O19175	Casein kinase I isoform alpha (Fragment) OS = Sus scrofa GN = CSNK1A1 PE = 2 SV = 1 - [KC1A_PIG]	CSNK1A1	51.13	1	−0.44	0.0473	Protein kinase activity
N0E654	Casein kinase II b subunit splicing isoform 476 (Fragment) OS = Sus scrofa GN = Csnk2b PE = 2 SV = 1 - [N0E654_PIG]	Csnk2b	63.97	2	−0.27	0.0039	Cell proliferation and cell differentiation

^a^Uniprot_ Sus scrofa_9823 database accession number

^b^The name of the protein exclusive of the identifier that appears in the database

^c^The sum of the scores of the individual peptides

^d^The number of distinct peptide sequences in the protein group

^e^Differential protein expression in the treatment group was presented as a log_2_ fold change relative to the control group

### GO annotations of proteins of differential abundance

**Fig. 2:**
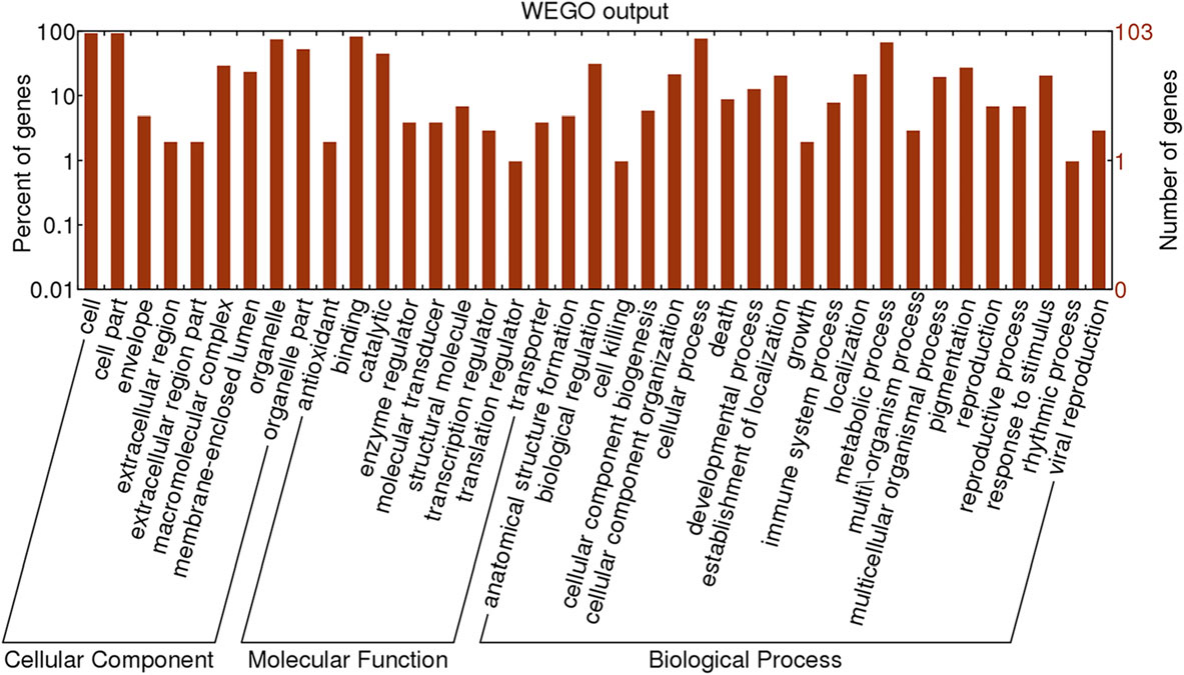
GO distribution analysis of differentially expressed proteins in small intestinal mucosal samples from the NSPE group and control group. The right coordinate axis indicates the number of proteins for each GO annotation, and the left one represents the proportion of proteins for every GO annotation

In the cellular component group, the differentially expressed proteins were concentrated in the intracellular part and membrane-bounded organelles (Fig. 2). In the molecular functional group, the differentially expressed proteins that are binding proteins (protein, nucleotide, or nucleic acid binding) and metabolic enzymes (hydrolase, oxidoreductase, or transferase activity) were ranked at the top of the category (Fig. 2). In the biological process category, the proteins that participate in cellular process (organelle organization process), metabolic process (nitrogen compound metabolic and biosynthetic process), and biological regulation (transcriptional and translational regulation, redox homeostasis, and immune response) had the highest ratios among the differentially expressed proteins.

### Validation of proteins of differential abundance

**Fig. 3:**
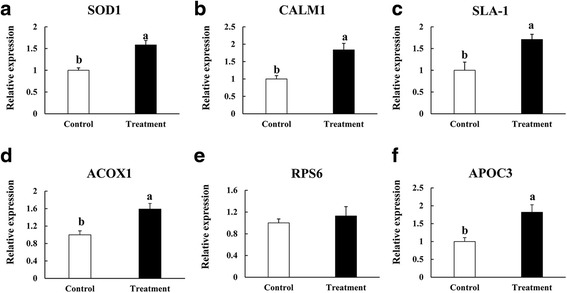
qPCR validation of six proteins of differential abundance from the intestinal mucosa of growing pigs at the mRNA level (**a**, **b**, **c**, **d**, **e** and **f**). Samples were normalized with the reference gene β-actin. Vertical lines represent means ± S.D, and different letters denote significant difference at *P* < 0.05 (*n* = 4)

Six differentially expressed proteins superoxide dismutase (SOD1) involved in redox homeostasis; calmodulin (CALM1) involved in calcium ion binding; MHC class I antigen (SLA-1) involved in immune response; acyl-coenzyme A oxidase (ACOX1) involved in energy metabolism; 40S ribosomal protein S6 (RPS6) involved in transcriptional and translational regulation; and apolipoprotein C-III (APOC3) involved in lipid absorption, were selected for the validation of proteomic data at the mRNA level using qPCR (Fig. 3). Most protein levels were consistent with their mRNA expression levels, except for RPS6.

## Discussion

The benefit of NSPEs supplementation is well recognized in monogastric animal production; NSPEs supplementation promotes growth performance and GI tract health, including the efficiency of nutrient utilization [2, 3, 8]. A number of studies have proven that the addtion of NSPEs to the diet reduces digesta viscosity by the partial or complete hydrolysis of soluble NSPs, which triggers the changes in microbial composition, especially the reduction of the amount of pathological bacteria within the small intestine [11, 32]. Moreover, the supplementation of NSPEs could increase the nutrient availability in the intestinal lumen (for example, energy substrates and proteins) [12, 33]. All above effects of NSPE supplementation are due to the improvements of the intestinal environment. However, it is still largely unknown how the small intestinal mucosa of the hosts responds to alterations in the luminal environment triggered by the addition of NSPEs. The present study marks the first time that the well-established quantitative iTRAQ label-based technology was applied for the proteomic analysis of the small intestinal mucosa of growing pigs with dietary supplementation of NSPEs. Various functional groupings of differentially expressed mucosal proteins related to nutrient metabolism, transcriptional and translational regulation, immune, and redox homeostasis were identified in response to NSPEs.

In former research, the utilization of β-glucanase and xylanase in the diet demonstrated that enzymes tended to increase the absorptive area and reduce cell proliferation and intraepithelial lymphocytes in the gut of pigs [34]. Both cereal grains and enzymes would affect components of gut health, including intestine morphology, bacteria populations, and microbial metabolites in the gut content [35]. It has been demonstrated that enhanced cell proliferation in the intestinal mucosa is associated with bowel diseases, cellular repair, and apoptosis [36, 37]. As shown in the present study, 89% of proteins related to transcriptional and translational regulation were down-regulated in NSPE pigs. We speculate that supplementation with NSPEs in the diet of growing pigs can reduce the possibility of intestinal infection. This is consistent with the former research result that NSPEs reduce the amount of pathological bacteria within the small intestine by lowering the viscosity of intestinal digesta [11].

The abundance of proteins CFL1 (cofilin-1), CFL2 (cofilin-2) and ARPC5 (actin-related protein 2/3 complex subunit 5), which are classified as cell cytoskeleton proteins relevant to cell structure and mobility, was increased. CFL1 and CFL2 are widely distributed intracellular actin-modulating proteins [38]. These two proteins can cause actin cytoskeleton rearrangement and membrane remodeling to the formation of phagosomes, which are recognized by Fc gamma receptors and beneficial for the host-defense in animals [39]. ARPC5 has a similar function as cofilin in the actin cytoskeleton, which is required for phagocytosis in mammals [40]. The up-regulation of these proteins might reflect the improved integrity of the intestinal mucosa.

As an important immune organ, the small intestine participates in the inflammatory response and the prevention of bacterial infection. SLA-1 (MHC class I antigen), GALNS (N-acetylgalactosamine-6-sulfatase), and DAI (DNA-dependent activator of IFN-regulatory factor) are considered to be involved in the immune response. SLA-1 alerts the immune system to virus-infected cells by presenting peptide fragments derived from intracellular proteins [41]. GALNS is located in lysosomes that digest different types of molecules and engulf viruses or bacteria within cells [42, 43]. DAI selectively enhances the DNA-mediated induction of type I IFN and other genes involved in innate immunity [44, 45]. The abundance of these proteins was up-regulated in NSPE pigs, suggesting that the supplementation of NSPEs may improve potential immunity and reduce the chance of bacterial infection in the small intestine. This is consistent with the elevated serum level of IgG in the NSPE group. However, challenges with exogenous pathogens are still required to verify the effect of NSPEs supplementation on immunity. In contrast, proteins involved in an inflammatory response, including NLRP6 (NLR family, pyrin domain containing 6) and CNPY3 (protein canopy homolog 3), are down-regulated, which indicates that inflammation is attenuated in the small intestinal mucosa due to the supplementation of NSPEs [46]. It has been suggested that one of the performance improvement attributes of NSPEs is due to the reduced local inflammation by controlling pathogens within the small intestine [32].

In addition to affecting the immune response, the up-regulated proteins catalase (CAT), glutaredoxin (GRXS), thioredoxin (TRX), superoxide dismutase (SOD), dimethylaniline monooxygenase [N-oxide-forming] 1 (FMO1) and 4-hydroxyphenylpyruvate dioxygenase (HPPD) are classified as redox homeostasis and detoxification proteins based on their primary functions. The up-regulation of CAT, GRXS, TRX and SOD may suggest that NSPE pigs had more potential to keep redox homeostasis in vivo [47–52]. This is consistent with the increased serum level of T-SOD in the NSPE group of this study. The reason for the up-regulation of these oxidoreductases and immune factors in the present study may be the increased abundance of reactive oxygen species (ROS) and inflammatory factors during stimulating energy metabolism due to a higher uptake of nutrients with NSPEs supplementation. However, further study is required to prove the effect of NSPEs on redox homeostasis. As one of the detoxification enzymes, FMO1 is regulated by xenobiotics, as the enzyme activity markedly increases in response to the invading harmful chemicals [53]. The up-regulation of this protein suggests that the supplementation of NSPEs is helpful to eliminate xenobiotics in the small intestine, which also could be related to the improvement of the intestinal lumen due to NSPEs.

Furthermore, the up-regulated abundance of proteins was observed in the NSPE group, including multiple nutrient metabolism processes such as energy, lipid, amino acid and mineral. These proteins included phosphoglycerate kinase 1 (PGK1), diazepam binding inhibitor (DBI), and acyl-coenzyme A oxidase (ACOX1). PGK1 plays a vital role in glycolysis or gluconeogenesis [54]. The up-regulation of ACOX1 indicates the elevation of glucose synthesis in the small intestine, which is consistent with the increased serum glucose level in the NSPE group. Likewise, higher abundance of DBI and ACOX1 was observed in this study, suggesting the stimulation of lipids β-oxidation for nutrient absorption to meet the energy requirement in the small intestine of NSPE pigs [55, 56]. Apolipoprotein C-III (APOC3) is an important modulator that is secreted from the intestine on the chylomicron upon lipid absorption [57]. The up-regulation of APOC3 implies the enhanced absorption of dietary lipids in the NSPE group.

Two differentially expressed proteins related to the permeability of the tight junction (TJ), including casein kinase II beta subunit splicing isoform 476 (Csnk2b) and myosin-4 (MYH4), were identified in the present study. The tight junctions (TJs) in the small intestine are not only a physical and biological barrier but also a passive diffusion system that depends on the permeability of the TJs [58]. Paracellular transport is one of the passive diffusion systems providing an absorption way for small molecular compounds [59], which are regulated by the permeability of the TJs and are thought to be important for mineral absorption [60]. Additionally, the transepithelial transport of oligosaccharides, but not polysaccharides, also occurs *via* the paracellular pathway [61]. Previous research has demonstrated that NSPEs are capable of hydrolyzing polysaccharides from the food to oligosaccharides in the gut [62]. Thus, the down-regulation of these two proteins in this study, in addition to former studies, indicates an increased permeability of the TJs in the NSPE group, which is beneficial to small molecular compounds absorption in the small intestine.

Calmodulin regulates cellular calcium concentration as a primary calcium-binding protein [63]. Calcium absorption is reduced if the bioavailability of dietary calcium is lowered by calcium-binding agents like cellulose because nearly all dietary calcium intake occurs in the upper intestine [64]. The up-regulation of this protein observed in this study suggests that calcium absorption in the small intestine is facilitated in the NSPE group by the degradation of calcium-binding agents in the diet, which could be conductive to bone health.

It has been demonstrated that one of the important roles of NSPEs within the small intestine is the elimination of the nutrient-encapsulating effect of cell wall polysaccharides, which increases the availability of starches, amino acids, and minerals. These results are consistent with our results from the present study that the levels of proteins related to nutrient absorption and utilization (energy, lipid, amino acid and mineral) are up-regulated. A fully understanding of the mechanisms of NSPEs supplementation will require the determination of protein modifications and protein regulation such as phosphorylation or glycosylation [65]. However, this part was not involved in the present study due to the technical limitation. Thus, further study is required to prove the effect of NSPEs on regulatory proteins using specific method, for example, the phosphoproteome.

## Conclusions

The results of this study provide the first evidence that the small intestinal mucosa proteome is altered in growing pigs supplemented with NSPEs. Growing pigs most likely responded to the increased reactive oxygen species (ROS) and inflammatory factors during stimulating energy metabolism due to NSPEs supplementation by changing the abundance of certain mucosal proteins that modulate redox homeostasis and enhance immune response. Most important of all, the effect of NSPEs on the increase of nutrient availability in the intestinal lumen provided additional benefits to facilitate protein expressions related to the efficiency of nutrient absorption and utilization, such as energy metabolism, amino acid metabolism, mineral metabolism, lipid absorption, and cell structure and mobility. These novel findings show the mechanisms whereby dietary supplementation with NSPEs promotes growth performance and improves the GI health of growing pigs, which also has important implications for the better utilization of this feed additive.

## Additional files

Additional file 1: The detailed description of the experiment methods, including mass spectrometric analysis procedures and parameters, bioinformatics analysis softwares, websites and real-time qPCR procedures. (DOCX 15 kb)
Additional file 2: Table S1. qPCR primers used for verification of the differentially expressed genes of the small intestinal mucosa in growing pigs. (DOCX 14 kb)
Additional file 3: Table S2. List of all proteins (*n* = 2634) identified in the study. (XLSX 199 kb)
Additional file 4: Table S3. List of all differentially expressed proteins (*n* = 104) identified in the study. (XLSX 31 kb)

## Abbreviations

ACOX1: Acyl-coenzyme A oxidase
ADFI: Average daily feed intake
ADG: Average daily gain
ALP: Alkaline phosphatase
ALT: Alanine aminotransferase
APOC3: Apolipoprotein C-III
ARPC5: Actin-related protein 2/3 complex subunit 5
AST: Aspartate aminotransferase
CALM1: Calmodulin
CAT: Catalase
CFL: Cofilin
CK: Creatine kinase
CNPY3: Protein canopy homolog 3
Csnk2b: Casein kinase II beta subunit splicing isoform 476 MYH4: myosin-4
DAI: DNA-dependent activator of IFN-regulatory factor
DTT: Dithiothreitol
FCR: Feed conversion ratio
FMO1: Dimethylaniline monooxygenase [N-oxide-forming] 1
GALNS: N-acetylgalactosamine-6-sulfatase
GI: Gastrointestinal
GRXS: Glutaredoxin
HEPES: 4-(2-hydroxyethyl)-1-piperazineethanesulfonic acid
HPPD: 4-hydroxyphenylpyruvate dioxygenase
IgG: Immunoglobulin G
iTRAQ: Isobaric tags for relative and absolute quantitation
NSPEs: Non-starch polysaccharide enzymes
PGK1: Phosphoglycerate kinase 1
PMSF: Phenylmethanesulfonyl fluoride
RPS6: 40S ribosomal protein S6
SLA-1: MHC class I antigen
SOD1: Superoxide dismutase
TP: Total protein
TRX: Thioredoxin
T-SOD: Total superoxide dismutase
