# Supplementary material for: Proteome changes in the small intestinal mucosa of growing pigs with dietary supplementation of non-starch polysaccharide enzymes
Source: Proteome Sci. 2017 Jan 10;15:3. doi: 10.1186/s12953-016-0109-6 (PMC5223414; doi:10.1186/s12953-016-0109-6)
Supplement: Additional file 1: — The detailed description of the experiment methods, including mass spectrometric analysis procedures and parameters, bioinformatics analysis softwares, websites and real-time qPCR procedures. (DOCX 15 kb) [file 12953_2016_109_MOESM1_ESM.docx]

**The detailed description of experiment methods**

**LC-MS/MS**

Each peptide fraction was re-dissolved in buffer A (2% acetonitrile, 0.1% formic acid) and centrifuged at 20,000 × g for 10 min. The final concentration of peptides in each fraction was approximately 0.25 μg/μl on average. Twenty microliter of supernatant was loaded onto an UltiMate 3000 Nano LC system (Bannockburn, IL) by the auto sampler onto a C18 trap column (length 2 cm, inner diameter 200 μm). Peptides were eluted onto a resolving analytical C18 column (length 10 cm, inner diameter 75 μM, 5-μm particles, 300 Å) packed in-house. Samples were loaded at 15 μl/min for 4 min and eluted with a 45-min gradient at 400 nl/min from 5 to 60% buffer B (98% acetonitrile, 0.1% formic acid), separated with a 3- min linear gradient to 80% B, maintained at 80% B for 7 min, and finally returned to 5% B over 3 min. The peptides were subjected to nanoelectrospray ionization followed by tandem mass spectrometry (Q-Exactive, Thermo) coupled online to the nanoLC. Intact peptides were detected in the Orbitrap at a resolution of 70,000 full with width at half maximum (FWHM). Peptides were selected for MS/MS using high-energy collision dissociation (HCD) operating mode with a normalized collision energy setting of 28%; ion fragments were detected in the Orbitrap at 17,500 FWHM resolution. A data-dependent acquisition mode that alternated between aMS scan followed by MS/MS scans was applied for the 10 most abundant precursor ions (2^+^ to 4^+^) above a threshold ion count of 20,000 in the MS survey scan with a following Dynamic Exclusion duration of 15 s (isolation window of m/z 2.0 and a maximum ion injection time of 100 ms). The electrospray voltage applied was 1.8 kV. Automatic gain control (AGC) was used to optimize the spectra generated by the Orbitrap. The AGC target for full MS was 3E6 and 1E5 for MS2. For MS scans, the m/z scan range was 350 to 2000. For MS2 scans, the m/z scan range was 100-1800.

**Database Search parameters**

MS/MS data for iTRAQ protein identification and quantitation were analyzed using Proteome Discover 1.3 (Thermo Fisher Scientific) and searched with in-house MASCOT software (Matrix Science, London, U.K.; version 2.3.0) against the database Uniprot_pig (Apr. 11th, 2014) with the following parameters: enzyme: trypsin; fixed modification: carbamidomethyl (C); variable modifications: oxidation (M), gln-pyroglu (N-term Q), iTRAQ 8-plex (N-term, K, Y); peptide mass tolerance:15 ppm; MS/MS tolerance: 20 mmu; maximum missed cleavages: 1. Identified peptides had an ion score above the threshold of peptide identity established by Mascot, and protein identifications were accepted within the false discovery rate (FDR) of 1% in which at least one such unique peptide match was specific for the protein.

**Bioinformatics analysis of proteins differential abundance**

Gene Ontology (GO) distribution for all of the proteins that were significantly altered in the small intestinal samples of growing pigs supplemented with NSPE were classified using Blast2GO software (<http://www.blast2go.com/>) and WEGO (http://wego.genomics.org.cn) that were provided by the Institute for Genomic Research.

**Real-time quantitative PCR (qPCR)**

Total RNA from intestinal mucosal samples was isolated using a Qiagen RNeasy Plus Mini Kit (Valencia, CA). The quality of the RNA was evaluated by electrophoresis on an agarose gel, and the quantity of the RNA was measured with a spectrophotometer (Nanodrop 2000, Thermo Scientific, Waltham, MA).

Reverse transcription was performed immediately following total RNA isolation using PrimeScript™ Reverse Transcriptase, D2680A (Takara, Dalian, China). RT-qPCR was performed using an Applied Biosystems 7500 Fast Real-Time PCR System (Foster City, CA). RT-qPCRs were performed at 95 °C for 30 s, followed by 40 cycles of 95 °C for 10 s and 60 °C for 30 s. SYBR green fluorescence was detected at the end of each cycle to monitor the amount of PCR product. A standard curve was constructed using a 10-fold dilution series, and its slope was used to calculate the efficiency of the qPCR primers. Primer sequences are listed in Table S1.

The expression level of a target gene mRNA was normalized to the mRNA level of β-actin. The ΔΔCT was calibrated against an average from the control group. The linear amount of the target gene expression to the calibrator was calculated by 2^−ΔΔCT^. Therefore, all gene expression results are reported as the fold difference between treated and control groups. The specificity of the real-time PCR product was verified using a melting curve and DNA sequencing.
