# Supplementary material for: Proteome changes in the small intestinal mucosa of growing pigs with dietary supplementation of non-starch polysaccharide enzymes
Source: Proteome Sci. 2017 Jan 10;15:3. doi: 10.1186/s12953-016-0109-6 (PMC5223414; doi:10.1186/s12953-016-0109-6)
Supplement: Additional file 2: Table S1. — qPCR primers used for verification of the differentially expressed genes of the small intestinal mucosa in growing pigs. (DOCX 14 kb) [file 12953_2016_109_MOESM2_ESM.docx]

**Table S1 The qPCR primers used for verification of the differentially expressed genes of the small intestinal mucosa of growing pigs**

| Gene name | Primer sequence (5'→3') | Product length（bp） |
| --- | --- | --- |
| β-actin | forward: CCACGAAACTACCTTCAACTC  reverse: TGATCTCCTTCTGCATCCTGT | 131 |
| SOD1 | forward: GTGCAGGGCACCATCTACTT  reverse: TTTGCCAGCAGTCACATTGC | 231 |
| CALM1 | forward: GACAGCGAGGAGGAAATCCG  reverse: CAGCTTCTCACCCAGGTTCG | 111 |
| SLA-1 | forward: TAGAGAAGGAGGGGCAGGAC  reverse: ACTTGCGCTTGGTGATCTGA | 285 |
| ACOX1 | forward: ATAAGGGAGTTTGGCCTCGC  reverse: TCCTGCTGTTCCTCAGTTGC | 143 |
| RPS6 | forward: CGAGAACTGGAAGCGCCTAT  reverse: CACCCTGCTTCATGGGGAAT | 287 |
| APOC3 | forward: GGACACCTCCCTTCTGGACA  reverse: GTTCGGGCTTGGGGGTATAA | 206 |

SOD1 = superoxide dismutase; CALM1 = calmodulin; SLA-1 = MHC class I antigen; ACOX1 = acyl-coenzyme A oxidase; RPS6 = 40S ribosomal protein S6; APOC3 = apolipoprotein C-III.
